# An investigation of inequalities in exposure to PM_2*.*5_ air pollution across small areas in Ireland

**DOI:** 10.1186/s12942-024-00377-4

**Published:** 2024-07-06

**Authors:** Aislinn Hoy, Gretta Mohan, Anne Nolan

**Affiliations:** 1 Economic and Social Research Institute, Whitaker Square, Sir John Rogerson’s Quay, Dublin 2, D02 K138 Ireland; 2 Department of Economics, Trinity College, Dublin, Ireland

**Keywords:** Air pollution, Health inequalities, Health policy, Particulate matter (PM_2*.*5_)

## Abstract

The link between exposure to air pollution and adverse effects on human health is well documented. Yet, in a European context, research on the spatial distribution of air pollution and the characteristics of areas is relatively scarce, and there is a need for research using different spatial scales, a wider variety of socioeconomic indicators (such as ethnicity) and new methodologies to assess these relationships. This study uses comprehensive data on a wide range of demographic and socioeconomic indicators, matched to data on PM_2.5_ concentrations for small areas in Ireland, to assess the relationship between social vulnerability and PM_2.5_ air pollution. Examining a wide range of socioeconomic indicators revealed some differentials in PM_2*.*5_ concentration levels by measure and by rural and urban classification. However, statistical modelling using concentration curves and concentration indices did not present substantial evidence of inequalities in PM_2*.*5_ concentrations across small areas. In common with other western European countries, an overall decline in the levels of PM_2*.*5_ between 2011 and 2016 was observed in Ireland, though the data indicates that almost all small areas in Ireland were found to have exceeded the World Health Organization (WHO)’s PM_2*.*5_ annual guideline (of 5 µg*/*m^3^), calling for greater policy efforts to reduce air pollution in Ireland. The recent Clean Air Strategy contains a commitment to achieve the WHO guideline limits for PM_2*.*5_ by 2040, with interim targets at various points over the next two decades. Achieving these targets will require policy measures to decarbonise home heating, promote active travel and the transition to electric vehicles, and further regulations on burning fossil fuels and enforcing environmental regulations more tightly. From a research and information-gathering perspective, installing more monitoring stations at key points could improve the quality and spatial dimension of the data collected and facilitate the assessment of the implementation of the measures in the Clean Air Strategy.

## Introduction

In an effort to improve overall population health, reducing health disparities based on social factors such as education, race, and socioeconomic status has emerged as a key objective for governments, policymakers, and international bodies such as the World Health Organization (WHO) [1]. Concurrently, understanding the role of social determinants of health has attracted increasing consideration in health research and academic literature. Social determinants of health are the conditions people encounter daily, including the places, environment, and circumstances in which they are born, grow, work, and live. Social determinants of health also encompass the broader forces and systems around these conditions. Previous literature has found that groups characterised by lower socioeconomic status typically have poorer health than more advantaged groups [1–3] and that differences in exposure to conditions that promote or, on the other hand, harm, human health can give rise to unequal and unjust health outcomes for different social groups [4]. When considering environmental risk factors, research has also found that socially disadvantaged groups can be disproportionately exposed to health-damaging characteristics of their physical environments, including air pollution [5]. Therefore, it has been suggested that not only do more socio-economically deprived groups have higher exposure risk, but they are also more vulnerable to the health-damaging effects of environmental exposures [6].

Ambient air pollution poses one of the most significant environmental threats to human health, contributing to reduced lung function, cardiovascular disease risk, increased cancer risk, and mortality [5, 7–9]. In particular, fine particulate matter, PM_2*.*5,_ has been identified as one of the most damaging types of air pollution for human health as the particles can easily infiltrate the blood and cardiovascular systems, increasing the risk of mortality [4]. In 2022, 97% of urban populations in the European Union (EU) were exposed to levels of PM_2*.*5_ concentrations that exceeded the health-based guidelines set by the WHO (of 5 µg/m^3^) [10]. Further, in 2020, urban concentrations of PM_2*.*5_ were estimated to have led to 238,000 premature deaths across the EU-27 [10].

Internationally, Ireland compares favourably for air pollution levels, as shown by the 8th lowest ranking of PM_2*.*5_ concentrations in 2022 of 37 European reporting countries [10]. According to the Environmental Protection Agency Ireland, PM_2*.*5_ levels have consistently been below EU policy limits but above WHO guidelines [4, 11]. The primary source of PM_2*.*5_ in Ireland is solid fuel burning for home heating. This leads to concern about local exceedances of health guidelines, particularly in winter. There are multiple domestic policies in Ireland tackling air quality, including bans on specific pollutant use, taxes on high-polluting vehicles and public health policies. A ‘*smoky coal*’ ban was gradually introduced between 1990 and 2022 [12, 13]. This legislation banned the burning of smoky coal and, in October 2022, further banned the commercial sale of peat or wet wood, significant contributors to air pollution [14]. The *Clean Air Strategy for Ireland* sets out targets to achieve the WHO air quality guidelines, including annual PM_2*.*5_ concentration levels below 5 µg/m^3^ by 2040 [15]. In terms of broader health policy, one of the four key goals of the *Healthy Ireland* policy is to reduce health inequalities, of which the outcomes frameworks set out to monitor air pollution progress [16]. The *Sláintecare framework* has set out to target 19 areas for intervention in reducing health inequalities. This has led to an increased focus on equality and environmental standards at the local level in recent years in Ireland [17].

Since socio-economically disadvantaged population groups may experience higher vulnerability and also more pronounced adverse health effects of air pollution, understanding whether there is a relationship between air pollution levels and the social and economic conditions of areas is of significant policy relevance. Where patterns are found, such evidence can be instructive for devising policy measures to address inequalities. For 2013–14, the European Environment Agency [5] examined inequalities in exposure to PM_2.5_ at the administrative levels of NUTS-2 and NUTS-3 (Nomenclature of Territorial Units for Statistics), which approximate to regional or city levels, across European Union Member States. While these units provide more detailed information at smaller scales than country-level, the units of analysis are relatively coarse, and studies of population health and local health risks at finer spatial scales are needed to help to understand the local situation and provide a useful source of information for local decision-makers [18–20]. Consequently, this paper is motivated to assess the association between living in areas of different socio-economic compositions and corresponding air pollution levels at the most granular spatial level available for Ireland, Small Areas. Based on the findings of international literature to date, it may be hypothesised that small spatial areas characterised by lower socioeconomic status indicators or higher deprivation scores experience greater PM_2*.*5_ air pollution [5].

The study is intended to contribute to the international debate on the geographic dimension of health-related environmental exposures and guide policymaking and planning in Ireland and internationally. This paper also addresses a gap in the research literature by exploring the associations between air pollution and socio-economic characteristics for an island nation with relatively low levels of PM_2.5_ using more contemporary data from 2016 onwards (where existing evidence typically relates to European counties with data from the 1990s and early 2000s characterised by much higher levels of PM_2.5_). In particular, it contributes to the call by organisations such as the European Environment Agency [5], and researchers in population health, for further research using a variety of spatial scales, incorporating additional indicators of social vulnerability (such as ethnicity) and using different methodologies to assess inequalities in exposure to air pollution and other environmental hazards [5, 18–20]. The remainder of this paper is structured as follows. The subsequent section discusses the literature on environmental inequality, followed by a description of the data and methods used for this analysis. The results are presented, and the implications of the findings are considered in the discussion section.

## Literature review

Environmental inequality refers to differences between socio-demographic groups’ exposure to environmental health hazards. Environmental inequality initially became a policy topic of interest during the United States (US) race movements of the early 1990s. The methodology of the academic literature that has emerged on this subject provides a structure for an overview of the evidence, arranged according to three approaches: distance-based studies, unit-based investigations, and risk-exposure models [21].

### Distance-based approaches

Distanced-based studies examine the demographic features of areas that are a specified geographic distance from environmental hazards, such as toxic waste sites, landfills, and industrial facilities [21]. In a study based in Florida, Pollock and Vittas [22] found that African Americans were the predominant ethnicity of residents in proximity to toxic substances treatment, storage, and disposal facilities (TSDF). For Los Angeles County, Boer et al. [23] also found that the communities most affected by TSDFs were African-American and Latino working-class communities near industrial areas. Chakraborty and Armstrong [24] linked Iowa census data to toxic release inventory (TRI) databases and concluded that areas with more TRIs were more likely to be inhabited by non-whites and people below the poverty level. Finally, a US national level study by Mohai and Saha [25] found that the percentage of lower socioeconomic groups and ethnic minorities decreased with increasing distance from TSDFs.

### Unit-based approaches

The unit-based approach, also known as the coincidence method, involves comparing the demographic characteristics of an area containing an environmental hazard site and the demographic characteristics of an area without an environmental hazard site [21]. Burke and Org [26] mapped industrial facilities that emitted toxic chemicals and linked them to demographic variables at census tract aggregated levels in Los Angeles, concluding that racial minorities were located in areas with more environmental hazard sites. Anderton et al. [27] used this spatial linkage method to combine mapped TSDF and US census data. Although they found that TSDFs were slightly more frequently located in areas with a more significant proportion of Hispanic people, they concluded no remarkable differences in ethnic composition between regions with established TSDFs. Oakes et al. [28] replicated this study, linking the same TSDF data set to related census data, finding no definite relationship between environmental risks and the communities in which racial and vulnerable groups were concentrated. Anderton et al. [29] further reported that the uncontrolled toxic facilities were not found to be disproportionately situated in poor or ethnic communities. This was challenged by Daniels and Friedman [30], who proposed that previous studies significantly underestimated the magnitude of racial disparities around hazardous waste facilities. Employing geographical information systems (GIS) approaches that better controlled for proximity, they mapped the 1990 US Environmental Protection Agency (EPA) toxic release inventory data set to US census tract data. The authors concluded that factors such as racial targeting and housing discrimination were associated with the location of waste facilities and that communities with a high proportion of black inhabitants were disproportionately exposed to toxic pollution.

In Australia, Chakraborty and Green [31] linked the spatial distribution of sites and emissions associated with industrial pollution to Indigenous status and social disadvantage characteristics of communities. A clear national pattern emerged, where communities with the most polluting sites, emission volume and toxicity-weighted air emissions had significantly greater proportions of Indigenous residents. Such sites were disproportionately based in regions and communities with the lowest educational attainment and occupational status levels. On the other hand, Lyons et al. [12] spatially examined the respiratory health of older people living in Ireland, comparing areas in which a ‘smoky coal’ ban was implemented (prohibiting the sale and use of ‘smoky coal’), and areas for which the regulation was not enacted, finding that for residents of areas which remained exposed to ‘smoky coal’, their respiratory health was worse than for those living in geographies subject to the ban.

### Risk-exposure approaches

Shao et al. [21] suggest that the unit-based and distance-based approaches to spatial analyses of environmental hazards are limited in quantifying health risks and instead advocate for the risk exposure model, which identifies toxic concentrations for geographical units. Ash and Fetter [32] combined 1990 US census block group data for urbanised areas with 1998 pollutants data adjusted for toxicity and dispersion levels to calculate exposure to air pollution. African Americans were found to reside in more polluted cities and more polluted neighbourhoods within cities, and those on lower incomes were significantly more exposed to pollution. Using air pollutant concentration data adjusted for toxicity and dispersion levels, Downey et al. [33] compared the environmental hazard burden experienced by ethnic minorities in US metropolitan areas. In line with Ash and Fetter [32], they concluded that African Americans often bore more considerable air pollution exposure than inhabitants of other ethnicities. These results have also been replicated in other studies from California [34] and the Midwest region [35]. In Canada, Buzzelli and Jerrett [36] used monitored air quality data and census data, finding that Asian Canadians were more greatly exposed to air pollution, and no clear correlation was uncovered for black Canadians.

In Europe, Forastiere et al. [37] analysed the relationship between area-based traffic emissions, income, and socioeconomic status of residents of Rome. They found more substantial particulate pollution in areas characterised by residents of lower socioeconomic status. Havard et al. [38] created a deprivation index from French census data, finding that mid-level deprivation areas were most exposed to nitrogen dioxide (NO_2_). For the Czech Republic, Branis and Linhartova [39] analysed differentials in exposure to Sulphur dioxide (SO_2_), NO_2_ and PM_10_ amongst urban populations according to educational level, unemployment rate, population size and average annual salary. They found differential associations according to city size; in larger cities, inhabitants with higher socioeconomic status were exposed to higher levels of traffic-related air pollutants, while the opposite pattern was observed for smaller cities.

Fernández-Somoano et al. [40] mapped air pollution as measured by NO_2_ concentrations and census data for Northern Spain, comparing urban and rural geographic units. The modelling revealed that NO_2_ was statistically significantly lower for census tracts with higher socioeconomic indices. However, a positive association between levels of education and NO_2_ exposure in urban areas was found, which did not occur in rural areas. In a cross-country comparison study, Fecht et al. [41] linked NO_2_ and PM_2.5_ exposure data to 2001 census data for small neighbourhood areas in England and the Netherlands. For both countries, neighbourhoods with more than 20% non-white residents had statistically significant positive associations with pollution exposure. For England, a similar relationship was found for areas where more than 20% of residents were not of working age (i.e., aged 0–14 and 65+). Finally, using the 2011 German census linked to pollution data, Rüttenauer [42] found that high-minority neighbourhoods were disproportionately affected by high levels of air pollution, especially within urban areas.

The range of studies from various jurisdictions report an array of findings that differ according to methodological approach and context. Most studies suggest that areas characterised by more socioeconomically disadvantaged populations are more likely to be exposed to environmental hazards [6]. There is a growing recognition of the need for studies at smaller spatial units to better understand within-area differentials in pollution exposures, of which air pollution presents one of the most significant health threats. In particular, a recent analysis of inequalities in exposure to air and noise pollution in Europe highlighted the need for further research at a variety of spatial scales, incorporating additional indicators of social vulnerability (such as ethnicity) and using different methodologies to assess inequalities in exposure to air pollution (and other environmental hazards) in European countries [10]. Building on the extant evidence, this study uses novel statistical techniques and comprehensive data on a wide range of demographic and socioeconomic indicators matched to data on PM_2.5_ concentrations at a small spatial scale in Ireland to assess the relationship between social vulnerability and PM_2.5_ air pollution.

## Materials and methods

### Spatial units of analysis

The unit of analysis in this study is the Irish Census 2016 Small Areas (SAs^1^). Small Areas (N = 18,641) are administrative regions developed by the Ordnance Survey Ireland (OSI) in collaboration with the Central Statistics Office (CSO). Unlike other Irish spatial units, SAs are defined by population rather than permanent geographic boundaries. On average, SAs contain 100 residences or 200 residents. As a result, SAs are relatively homogeneous in social composition and are the most nationally comparable Irish spatial unit. The SA shapefile can be downloaded from the CSO website.

### ***Air pollution measure- PM***_***2.5***_

The highest resolution maps (*1* km × *1* km) of annual mean concentrations of PM_2*.*5_ available for Ireland were sourced from the research team for the Data Integration Model for Air Quality (DIMAQ), developed by the WHO and the University of Exeter [42]. The DIMAQ model produces a comprehensive set of high-resolution estimates of concentrations of PM^43^_2*.*5_ from a combination of ground measurements, satellite, and chemical transport measurements, weighted by land use rates and local populations.^2^ Annual data is available for the period 1998–2020. In this study, we use data for 2016, which aligns with the Census of Population data collected in 2016 at the SA level. We also present data from an earlier year (2011) to show how PM_2*.*5_ levels have changed. The PM_2*.*5_ concentration data was aggregated to the SA level. The spatial distribution of the population within an SA was accounted for by averaging the concentration estimates of residential buildings in each SA and weighting by the number of addresses in each building. This corrects for the assumption that the population is spread uniformly across each SA.

### Deprivation, demographic and socioeconomic measures

This paper uses a selection of routinely available small area population statistics (SAPS) from the 2016 Census and the 2016 Haase–Pratschke (HP) deprivation index, a composite measure of area-level deprivation as measures of socioeconomic status.

#### Small Area Population Statistics

SAPS are the most granular available information on the demographic, social and economic composition of residents living in Ireland’s SA geographies as recorded for the usually resident population in the Irish 2016 Census. They are available from the CSO website and contain information for all 18,641 SAs. The SAPS provide the number of people in each SA with certain demographic, social and economic characteristics. The indicators analysed in this research are documented in Table 1, with a geographical depiction provided in Appendix A (Figure 8). For the analysis of this paper, we assign SAs a value equal to the proportion of their population with each of the demographic characteristics. We then divide the SAs into quintiles, where quintile one has the 20% of SAs with the smallest proportion of each indicator in question, while quintile five has 20% of SAs with the largest proportion.

**Table 1:** Description of small area population statistics

Indicator	Description
No third-level education	The proportion of the 15+ population in a SA without a third-level education (degree)
Non-working age population	The proportion of a SA aged between 0–14 years and above the age of 65
Non-owner-occupied housing	The proportion of the population in a SA that is residing in non-owner-occupied housing (e.g., rented accommodation)
Non-white ethnicity	The proportion of households in a SA that are non-white
Non-professional socioeconomic group	The proportion of the population in a SA of a non-professional occupation (by reference person)
Unskilled social class	The proportion of the population in a SA of an unskilled social class
Unemployed	The proportion of the 15+ population in a SA that is unemployed

#### Haase–Pratschke Deprivation Index

**Fig. 1:**
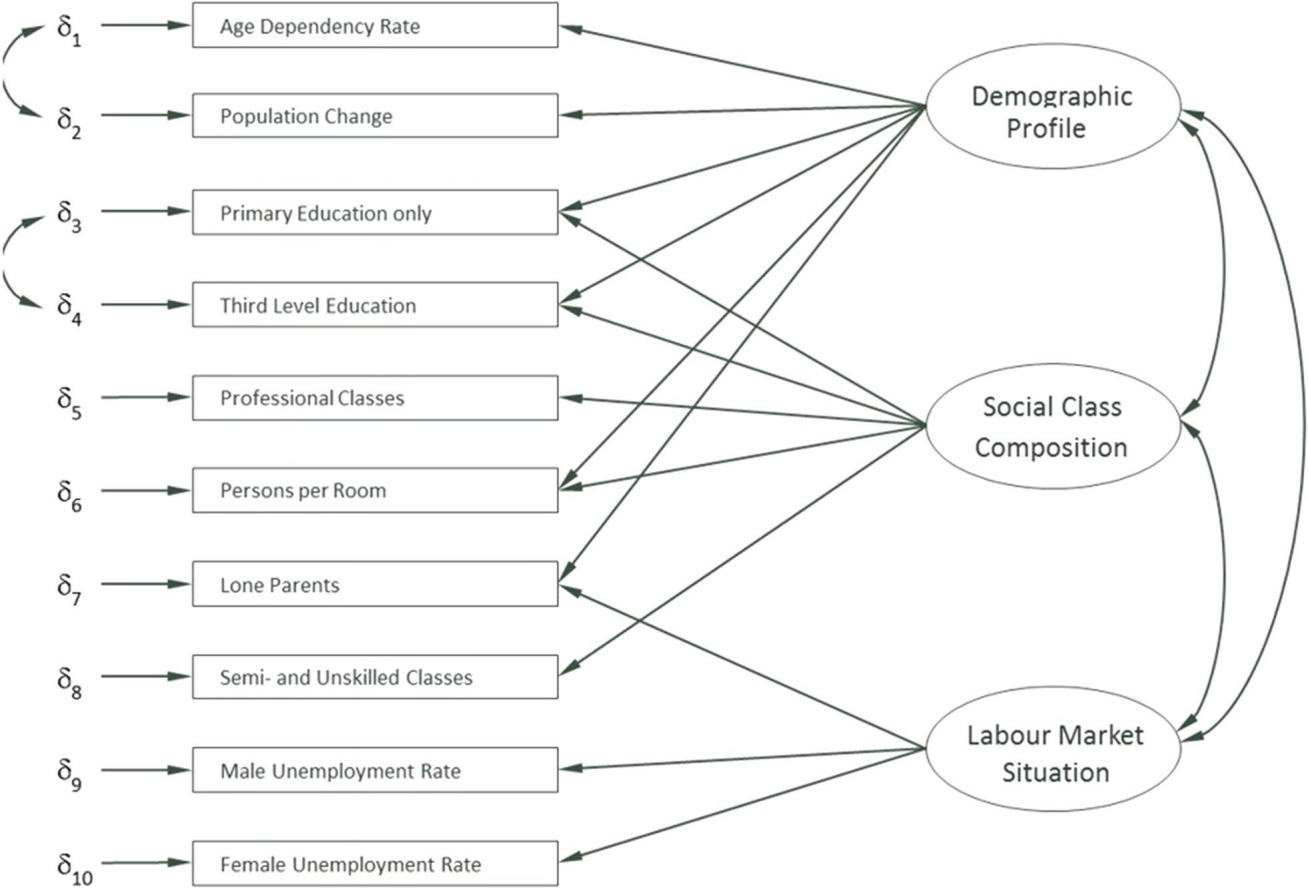
Basic model of the Haase–Pratschke (HP) Deprivation Index (Source: [54], p.4)

The HP index provides a metric for the deprivation level of SAs based on three main dimensions: demographic profile, social class composition and labour market conditions. While it resembles the UK’s indices of Multiple Deprivation [44], it is specifically tailored for the context of Ireland using information recorded in the Census, and is Ireland's most widely used social gradient metric. The scoring system ranges from − 41 (most deprived) to + 41 (most affluent), with zero being the national average. For Census 2016, the HP index assigned each of the 18,641 SAs an absolute deprivation score. We employ the HP index score for SAs itself in this study, as well as splitting the SAs into quintiles of deprivation created from the distribution of the HP scores. For this analysis, quintile five has the 20% of SAs which are the most affluent. The three dimensions of the HP index are calculated using a combination of SAPS indicators, where the structure of the information used to inform the index can be seen in Fig. 1 and additional information is detailed in Appendix B.^3^

#### Spatial linkage and assignment of urban classification

**Fig. 2:**
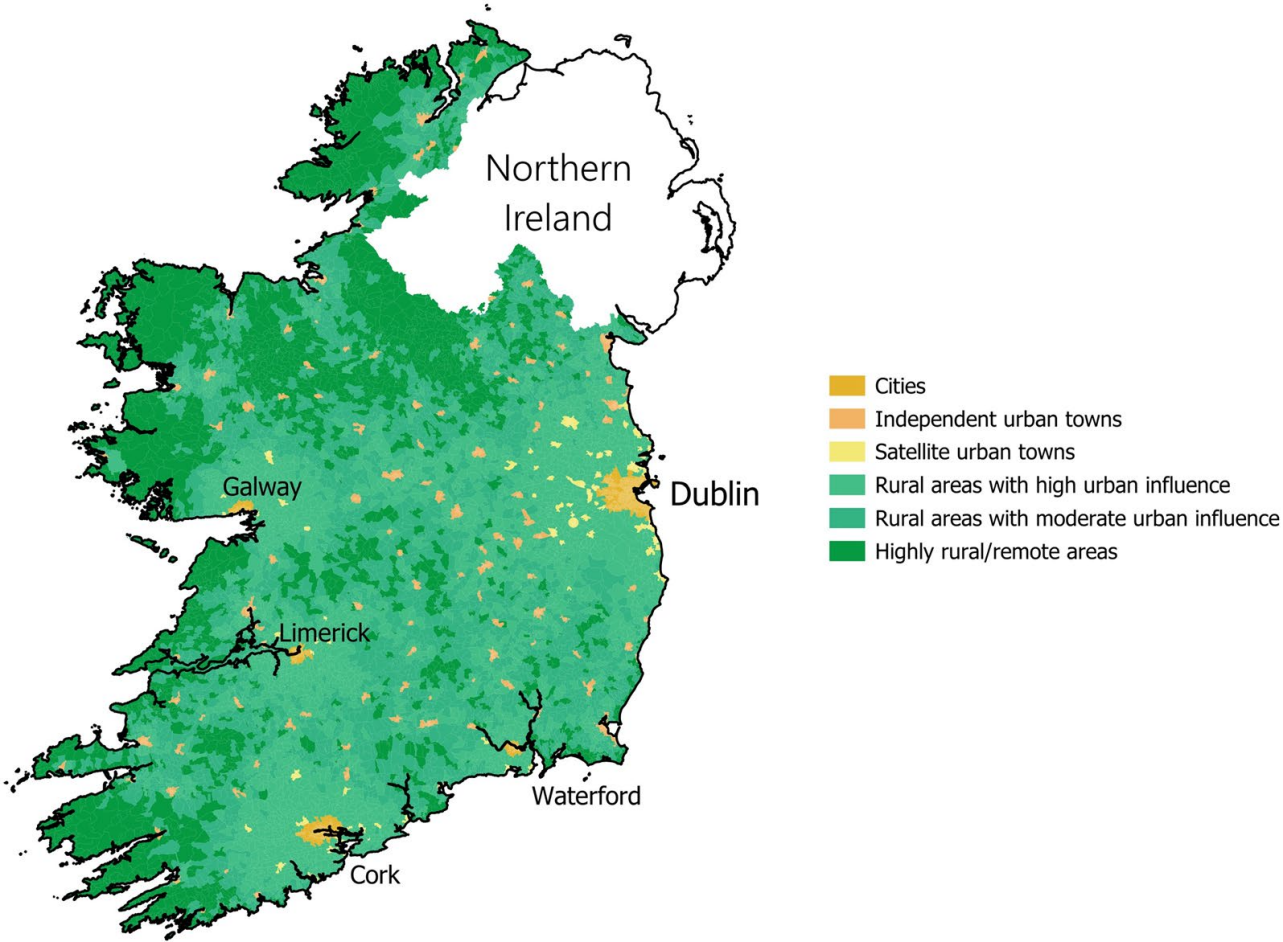
Small area urban rural classifications

A final data set was created using QGIS, assigning each 2016 SA an average PM_2.5_ concentration measurement (2011 and 2016), a HP index score, a HP index quintile, and SAPS indicator quintiles. The 2019 Urban and Rural Life in Ireland definitions were used to assign SAs to an urban or rural classification, illustrated in Fig. 2 ., [55].

### Analytical approach

PM_2*.*5_ concentration levels and the socioeconomic and demographic variables are initially described using summary statistics and maps. Quintile graphs of socioeconomic indicators by average annual concentrations of PM_2*.*5_ are presented at a national level and stratified by urban and rural status. Further, following Huang et al. [45], concentration curves are also used to explore the associations between PM_2*.*5_ concentration levels and socioeconomic and demographic variables. The concentration curves are calculated as follows:

$$C = \frac{1}{{\overline{y}}}cov\left( {y_{i} ,R_{i} } \right)$$

where y_*i*_ is the PM_2*.*5_ concentration of a SA, $\overline{y }$ is the mean level of y_*i*_ across SAs, and R_*i*_ is the SA ranked by the social or demographic variable. The population of an SA is ranked in descending order by the HP index or socioeconomic or demographic indicator on the horizontal axis. This is plotted against the corresponding cumulative PM_2*.*5_ concentration on the vertical axis. Concentration curves are plotted against a 45-degree line of equity. How the results are interpreted depends on the ranking of the variable. For the HP index, if the curve is below the equity line, SAs with a higher HP score (most affluent) are exposed to greater levels of PM_2*.*5_. If the concentration curve is above the equity line, SAs with a lower HP score (most disadvantaged) are exposed to greater levels of PM_2*.*5_. The HP variable is ranked inversely compared to the SAPS indicators; therefore, the interpretation of the HP index is the inverse of the SAPS indicators. When interpreting the SAPS indicators, if the curve is below the equity line, SAs with a higher percentage of the SAPS indicator (most disadvantaged) are exposed to greater levels of PM_2*.*5_. If the concentration curve is below the equity line, SAs with a lower percentage of the SAPS indicator (most advantaged) are exposed to greater concentration levels of PM_2*.*5_.

Concentration indices are derived from the concentration curves with values between − 1 and 1. For all SAPS indicators, a positive value indicates socioeconomic inequality in exposure to PM_2*.*5_, where those who are more advantaged experience lower levels of PM_2*.*5_ concentrations. Accordingly, a negative value indicates that those who are more advantaged experience higher levels of PM_2*.*5_ concentrations. The opposite is true for the HP index. All statistical analysis was performed using STATA 17.

## Results

### Descriptive statistics

Table 2 documents the summary statistics of the variables examined in this analysis (Tables 4, 5 and 6 in Appendix C provide additional information on the ranges and 95% Confidence Intervals). The average and maximum PM_2*.*5_ concentration levels declined from 2011 to 2016 but were still higher in urban areas than in rural areas. Rural areas were characterised by lower levels of education and higher proportions of the  non-working age population. Urban areas were more likely to have residents of non-white ethnicity and greater levels of non-owner-occupied households.

**Table 2:** Descriptive statistics

Variable	National				Urban	Rural
	Mean	Med	Min	Max	Mean	Mean
PM_2*.*5_ 2011	10.41	10.47	0.02	14.97	10.99	9.50
PM_2*.*5_ 2016	7.95	7.99	0.15	11.37	8.38	7.27
HP Index 2016	− 4.07	− 3.80	− 40.90	35.50	− 3.34	− 5.21
Census SAPS indicators						
No third-level education	0.70	0.73	0.00	1.00	0.66	0.75
Non-working age population	0.34	0.35	0.00	0.85	0.32	0.38
Non-owner occupied housing	0.30	0.22	0.00	1.00	0.40	0.16
Non-white ethnicity	0.05	0.02	0.00	0.700	0.08	0.01
Non-prof. socioecon.group	0.24	0.23	0.01	0.85	0.23	0.23
Unskilled social class	0.33	0.30	0.03	1.00	0.34	0.30
Unemployed	0.06	0.04	0.00	0.40	0.06	0.05

**Fig. 3:**
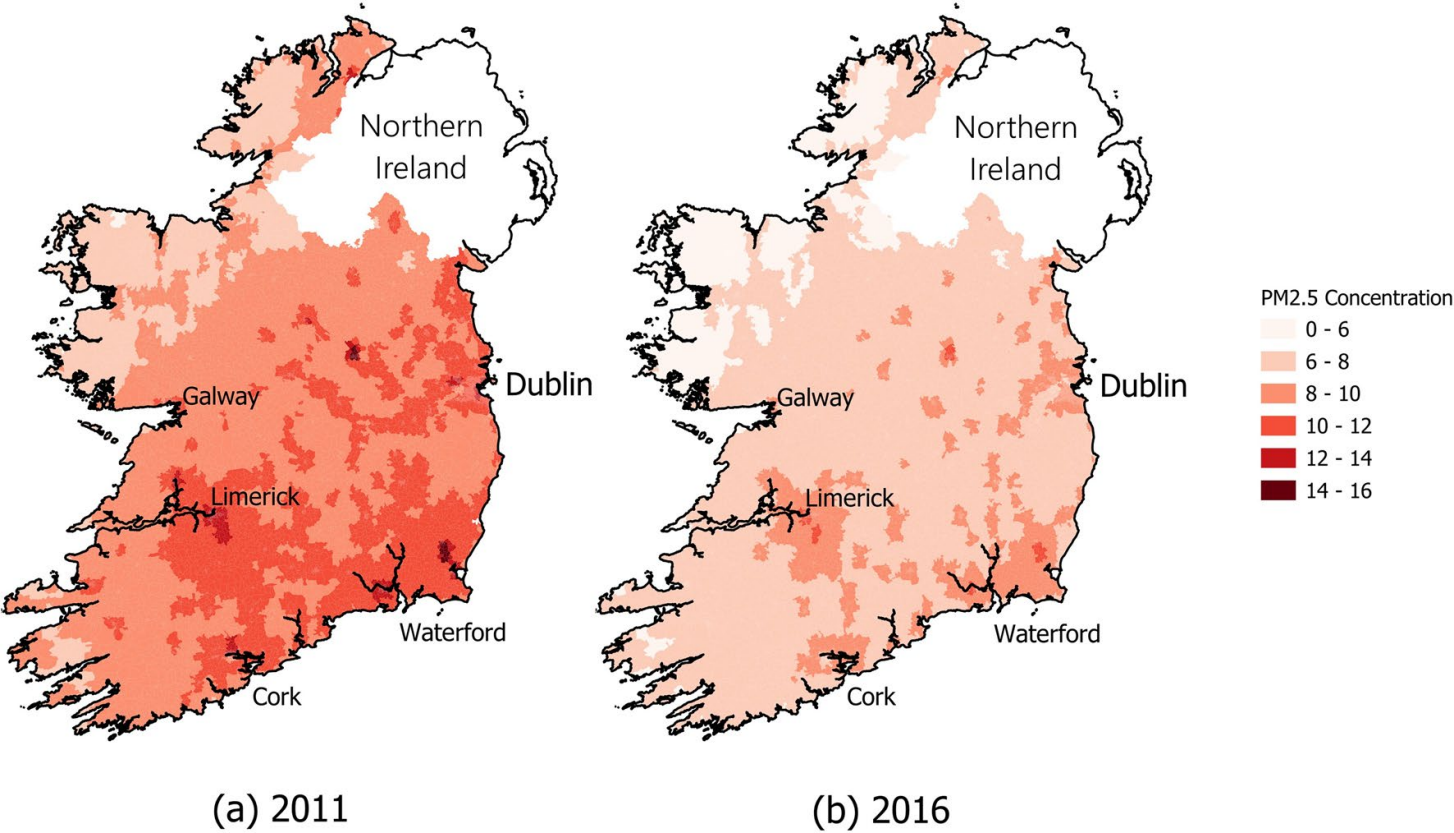
Average PM_2*.*5_ concentrations in SAs

The maps in Fig. 3 illustrate each SAs (n = 18,630)^4^ average PM_2*.*5_ concentration levels in 2011 and 2016. A significant improvement between the two periods is observable, with the mean PM_2*.*5_ concentration level decreasing by approximately 23% or 2.46 µg/m^3^. In 2011 and 2016, all SAs were within the annual legal limit of PM_2*.*5_ (25 µg/m^3^) as per the EU Ambient Air Quality Directive (2008/50/EC). However, more than 99% of SAs in both 2011 and 2016 exceeded the new annual guideline values of PM_2*.*5_ (5 µg/m^3^), set by the WHO [4], and this trend has continued post 2016 [11].

**Fig. 4:**
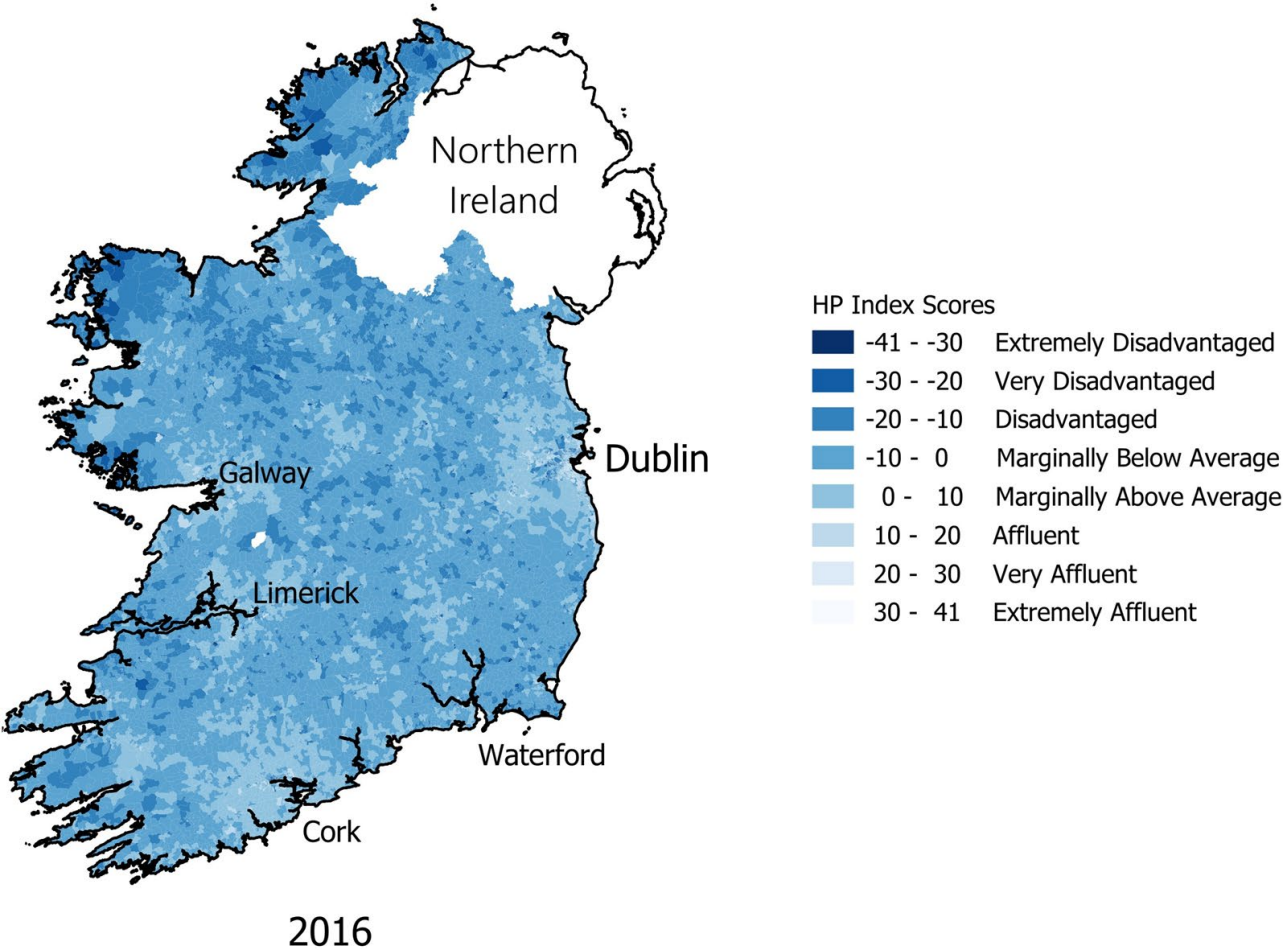
HP Index for Ireland 2016

Figure 4 depicts the HP Index for SAs in 2016 (n = 18,630). Affluence in Ireland is highest in Ireland’s urban peripheries, as can be seen around Dublin in the east and gradually declines towards rural locations.

**Fig. 5:**
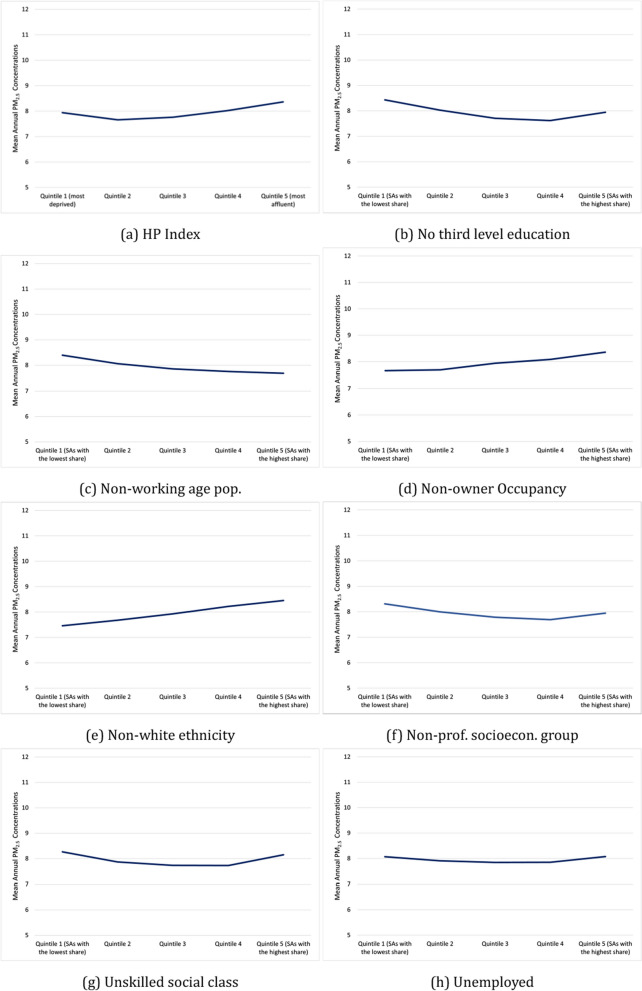
Average PM_2*.*5_ by share of the population

Figure 5 displays the mean annual PM_2*.*5_ exposure across the quintiles of area-level deprivation and the quintiles of the SAPS indicators. Focusing on the HP Index for 2016, a u-shaped relationship is observed, where the least and most deprived quintiles of SAs experienced the highest concentrations of PM_2*.*5_. In 2016, the most affluent quintile had an average annual PM_2*.*5_ concentration of 8.37 µg/m^3^, followed by the most deprived quintile with a concentration of 7.93 µg/m^3^. The middle-level deprivation quintiles had lower PM_2*.*5_ concentrations, with the lowest in quintile two.

When focusing on the SAPS indicators, several demonstrate a similar u-shaped relationship. These include no third-level education, unskilled social class, non-professional socioeconomic group, and unemployment. However, the differentials in concentrations across areas are minimal, with less than 1 µg/m^3^ difference between the foremost advantaged and most disadvantaged areas for these indicators. The non-white ethnicity and non-owner occupancy indicators display a weak but positive association with PM_2*.*5_ concentrations. This means that areas with a higher proportion of the population that is non-white or living in non-owner-occupied housing (i.e. renting) have higher average concentrations of PM_2*.*5_.

**Fig. 6:**
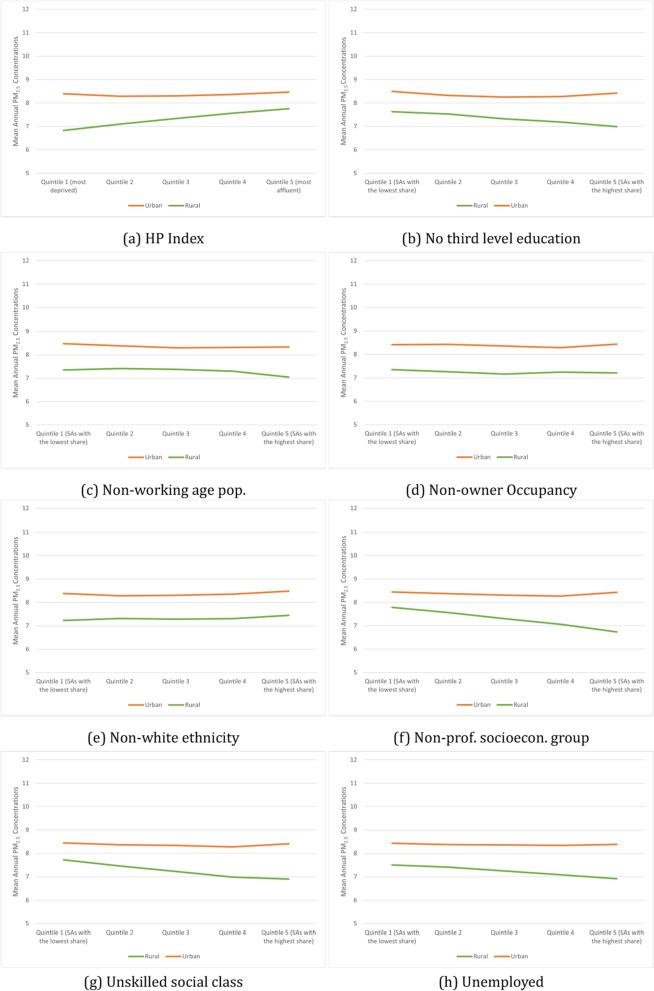
Average PM_2*.*5_ by share of the pop, stratified by urban and rural status

Figure 6 shows the mean annual PM_2*.*5_ concentrations across the quintiles of area-level deprivation and the quintiles of the SAPS indicators stratified by rural and urban areas.^5^ When considering the HP index, in urban areas, the difference in PM_2*.*5_ concentration levels across deprivation quintiles is minimal, with only a 0.17 µg/m^3^ difference between the quintile with the highest concentration (quintile five) and the quintile with the lowest concentrations (quintile two). On the other hand, the results highlight a positive relationship between mean annual PM_2*.*5_ concentrations and deprivation levels in rural areas. However, the concentration difference between the most and least deprived quintiles is still very slight (0.92 µg/m^3^). The data shows that the most affluent SAs in rural areas experience the highest PM_2*.*5_ concentration levels.

When considering the SAPS indicators, the difference in PM_2*.*5_ concentration levels across quintiles for any indicator is minimal in urban areas, with no clear positive or negative relationship. That is, no indicator has a difference greater than 1 µg/m^3^ between the quintile with the highest and lowest concentrations of PM_2*.*5_. In rural areas, there is also a minimal difference between quintiles for all SAPS indicators. However, they do, on average, show a negative relationship. Sub-populations based on education, social class, socioeconomic group, and unemployment all have the highest concentration rate in quintile one and the smallest share in quintile five.

**Fig. 7:**
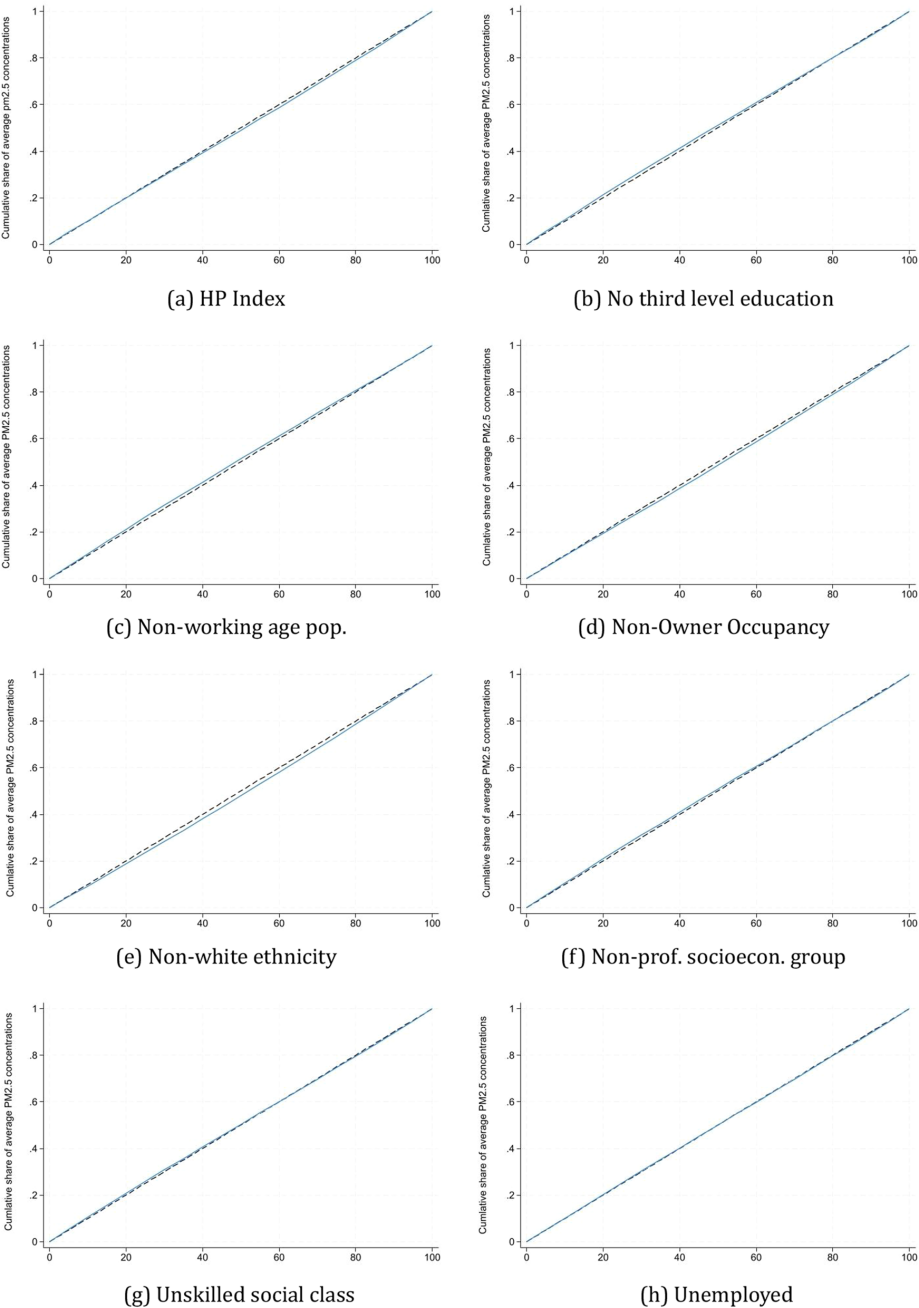
Concentration indices of PM_2*.*5_ concentrations by HP index and SAPS

**Fig. 8:**
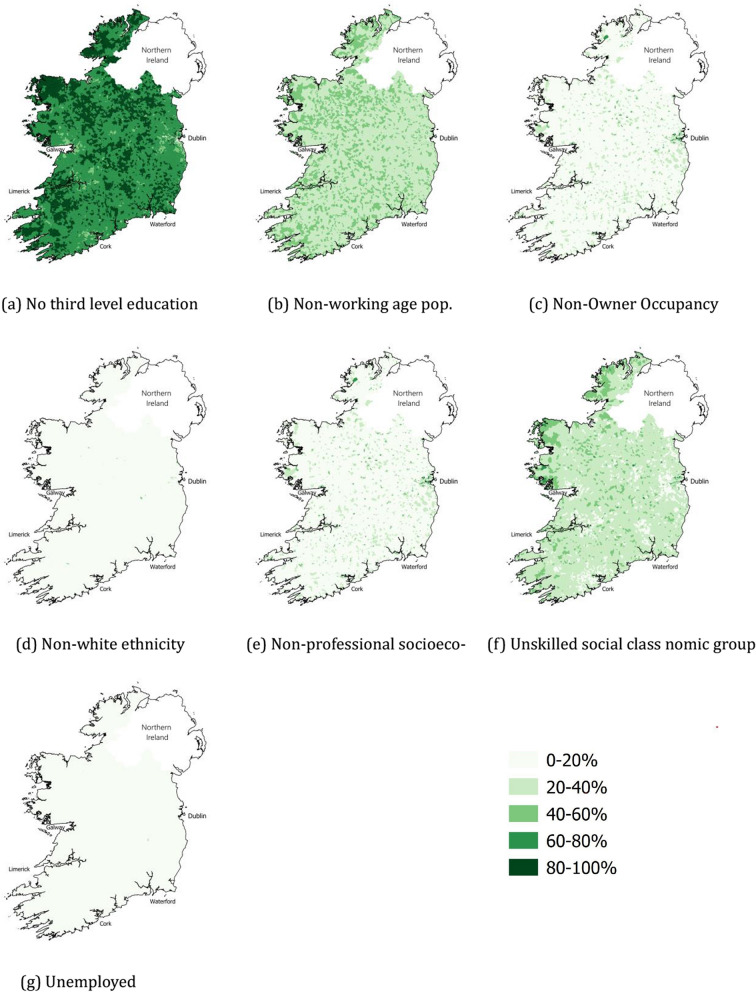
National maps of SAPS indicators

Figure 7 and Table 3 present the concentration curves and corresponding concentration indices. In 2016, the concentration index for the HP index PM_2*.*5_ exposure was 0.013. According to the sign, relatively affluent small areas had higher shares of PM_2*.*5_ concentrations. However, since a concentration index score of 0 indicates zero pollution-related inequality, the magnitude of the result indicates virtually zero inequality.^6^ The concentration indices were negative for sub-population groups based on no third-level education, non-working age population, unskilled social class, non-professional socioeconomic groups and unemployment. This suggests that areas with a higher average educational level, social class, profession, or employment status had higher shares of PM_2*.*5_ exposure. Although these estimates are statistically significant (p < 0.001), they are near zero (ranging from − 0.001 to − 0.018), indicating minimal pollution-related inequality by sub-populations. Two indicators are associated with a positive concentration index: non-white ethnicity and non-owner-occupied housing. This is interpreted that the higher the proportion of the population who are non-white or non-owner occupants (i.e. renting), the higher the shares of PM_2*.*5_ concentration in the small area. However, once again, the magnitude of the estimates is very small, which primarily reflects the minimal variation in PM_2*.*5_ levels across SAs in general, as illustrated in Figs. 3 and 5.

**Table 3:** Concentration indices

Indicator	Con. Index	Std. error	P-value
HP Index 2016	0.012	0.001	0.000
Census SAPS indicators			
No third-level education	− 0.014	0.001	0.000
Non-working age population	− 0.018	0.001	0.000
Non-owner-occupied housing	0.019	0.001	0.000
Non-white ethnicity	0.027	0.001	0.000
Non-prof. socioecon. group	− 0.010	0.001	0.000
Unskilled social class	− 0.004	0.001	0.000
Unemployed	− 0.001	0.001	0.632

## Discussion

The threat that air pollution presents to human health, combined with the potential for inequalities in exposure, merits thorough academic inquiry into the spatial, demographic, and socio-economic patterning of risks. The research presented in this paper uses linked census and PM_2*.*5_ data to analyse these issues at the smallest spatial scale for Ireland, a European country for which such an investigation has hitherto not been conducted. Encouragingly, the levels of air pollution observed have declined, though concerns remain as 99% of small areas in Ireland in both 2011 and 2016 were found to have exceeded the WHO’s PM_2*.*5_ guideline (5 µg/m^3^) [4]. The differences in PM_2*.*5_ exposure across demographic and socio-economic quintile groups were slight. Furthermore, minimal evidence of differences was detectable across SAs from the concentration curves and concentration indices. These findings collectively do not support the a priori hypothesis of this paper that more disadvantaged groups in Ireland encounter greater PM_2*.*5_.

This result may be explained by comparatively little heavy industry concentrations in Ireland [46], the gradual efforts to reduce air pollution across the country, including the phased roll-outs of a smoky coal ban across the country, and high wind levels due to the nature of Ireland being an island [12, 47]. Indeed, previous work has highlighted the fact that PM_2*.*5_ (in contrast to other forms of air pollution such as NO_2_) tends to vary much more over time than over spatial units, reflecting the fact that PM_2*.*5_ is heavily influenced by wind patterns [48, 49]. In this regard, future work replicating this analysis using alternative indicators of air pollution (for example NO_2_) will be important for assessing whether these findings concerning PM_2*.*5_ can be generalised to other forms of air pollution in Ireland.

In placing the findings of this work within the context of the extant literature, the lack of inequality in air pollution, as measured by PM_2.5_ concentrations, observed in the context of Ireland contrasts with other European literature, such as that reported by Fecht et al. [41] Rüttenauer [42] and Forastiere et al. [37]. The variation in results reported in the international literature by study setting and methodology suggests that the degree to which area-level demographics and PM_2*.*5_ level concentrations are associated appear context-specific. In this field of study, one cannot assume a particular direction of effect (or indeed a null effect) for any specific jurisdiction of interest [21], and for the reasons outlined above, including the island setting characterised by relatively strong winds, a low presence of heavy industry, and ongoing policy interventions to reduce air pollution, Ireland may not necessarily be representative of other countries.

We note that most studies have used specific demographic variables as proxies for determinants of socioeconomic status [50]. The most commonly investigated are ethnicity, income, and education level. More recently, particularly in the European context, studies have employed composite measures of deprivation [21, 38]. The research of this paper encompasses both of these approaches, analysing associations between the distribution of PM_2*.*5_, the HP index, and associations with seven socioeconomic variables. The associations between PM_2*.*5_ and the different socioeconomic indicators did not vary substantially, particularly for urban areas. In finding a similar result, Briggs et al. [50] explain that any single population demographic cannot readily characterise environmental inequities. Further, the authors suggest that a composite measure of these demographic indicators (such as the HP index) may be less accurate since it averages across the different indicators. The use of both specific indicators and a composite measure avoids generalising findings from one proxy deprivation socioeconomic variable to another. We note that for 27 EU countries, Richardson et al. [51] also reported a u-shaped pattern for PM_10_ levels, with the highest level in the lowest income quintile, lower values in quintiles two, three, and four, increasing again at quintile five.

Several limitations of this research must be acknowledged. Firstly, the environmental concentration data provided by DIMAQ is modelled over space and time. Since PM_2*.*5_ is positively correlated with solid fuel burning, by averaging ambient concentrations over space, these extremes are masked, and residents living in the same SA but using different home heating methods (and therefore potentially exposed to different indoor levels of air pollution) are modelled as experiencing the same ambient concentration values. Ambient (i.e., outdoor) concentrations do not take account of potential differences in personal exposure, due to differences in avoidance behaviour, indoor air pollution, housing conditions and daily activities. Our results, therefore, are subject to the ecological fallacy; group-level patterns cannot be assumed to translate into individual experiences. Still, given that within-area heterogeneity decreases as the size of the area decreases; our choice of spatial unit minimises this limitation considerably compared to other literature in the field [5]. Additionally, averaging the data to an annual measurement smooths variations of PM_2*.*5_ concentrations exposure between SAs, which may vary significantly depending on the season, especially considering home heating methods [52]. Access to daily data (for example, see Huang et al. [45], rather than annual averages, could allow for the creation of alternative models with finer temporal variation. Aguilar-Gomez et al. [56] discuss the trade-offs involved in using data on air pollution in empirical research, they note that in general, the finer the temporal scale, the coarser the spatial scale (and vice versa).

## Conclusion

This first Irish study of the spatial distribution of outdoor PM_2*.*5_, a health-harming substance found in air pollution, did not find strong evidence of inequities in exposure patterns across socioeconomic, demographic and deprivation indicators. Some nuanced patterns across the measures examined were observed, with associations differing slightly between urban and rural areas. While an overall decline in the levels of PM_2*.*5_ between 2011 and 2016 is a positive finding, the data indicates that almost all small areas in Ireland were found to have exceeded the WHO’s PM_2*.*5_ annual guideline (5 µg/m^3^), calling for greater policy efforts to reduce air pollution in Ireland. The recent *Clean Air Strategy* contains a commitment to achieve the WHO guideline limits for PM_2*.*5_ by 2040, with interim targets at various points over the next two decades. Achieving these targets will require policy measures to decarbonise home heating, promote active travel and the transition to electric vehicles, and further regulations on burning fossil fuels and enforcing environmental regulations more tightly. From a research and information-gathering perspective, installing more monitoring stations at key points could improve the quality and spatial dimension of the data collected and facilitate the assessment of the implementation of the measures in the *Clean Air Strategy*.

## Data Availability

Maps of annual mean concentrations of PM2.5 available for Ireland were sourced from the research team for the Data Integration Model for Air Quality (DIMAQ), developed by the World Health Organization and the University of Exeter (see Shaddick et al. 2021). This paper uses routinely available small area population statistics (SAPS) from the 2016 Census available from the Census Statistics Office in Ireland, see: https://www.cso.ie/en/census/. The 2016 Haase-Pratschke (HP) deprivation index, can be accessed from: http://trutzhaase.eu/services/hp_deprivation_index/.

## Appendices

### Appendix A: National maps of SAPS indicators

See Fig. 8.

### Appendix B: Explanation of the HP Deprivation Index

The deprivation index used in this paper is the Pobal Haas–Pratschke (HP) index of multiple deprivation [53]. This multiple deprivation index is created for the Republic of Ireland using census small data, and the deprivation scores are based on three dimensions of affluence/disadvantage: the demographic profile, social class composition, and labour market situation.

- *Demographic profile* is a measure of rural affluence/deprivation and is measured by six indicators [53]
  - the percentage change in population over the previous 5 years (positive association);
  - the percentage of the population aged under 15 or over 64 years of age (negative association);
  - the percentage of population with a primary school education only (negative association);
  - the percentage of population with a third level education (positive association);
  - the percentage of households with children aged under 15 and headed by a single parents (positive association);
  - the mean number of persons per room (positive association).
- *Social class composition* has a consider impact on many areas of life, and is a key factor in the inter-generational transmission of economic, cultural and social assets [53]
  - the percentage of the population with a primary school education only (negative association);
  - the percentage of the population with a third-level education (positive association);
  - the percentage of households headed by professionals or managerial and technical employees, including farmers with 100 acres or more (positive association);
  - the percentage of households headed by semi-skilled or unskilled manual workers, including farmers with less than 30 acres (negative association);
  - the mean number of persons per room (negative association).
- *Labour market situation* a predominantly urban measure. It accounts for the principal cause of disadvantage at a national level: unemployment [53]
  - the percentage of households with children aged under 15 and headed by a single parent (negative association);
  - the male unemployment rate (negative association);
  - the female unemployment rate (negative association).

### Appendix C: Descriptive statistics of PM2.5 concentrations by indicators for quintiles at the national level, and by rural and urban quintiles

See Tables 4, 5, 6.

**Table 4:** Descriptive statistics by quintiles at the national level

Variable	N	Mean	Min	Max	Lower 95% CI	Upper 95% CI
HP Index 2016						
Quintile 1	3656	7.93	4.35	11.39	7.89	7.97
Quintile 2	3683	7.65	0.79	11.38	7.62	7.69
Quintile 3	3682	7.76	0.19	11.36	7.73	7.79
Quintile 4	3665	8.00	1.00	11.36	7.97	8.03
Quintile 5	3609	8.36	0.15	11.37	8.33	8.38
No third-level education						
Quintile 1	3724	8.43	0.15	11.02	8.41	8.46
Quintile 2	3728	8.03	1.00	11.37	8.00	8.06
Quintile 3	3726	7.71	0.19	11.36	7.68	7.74
Quintile 4	3744	7.62	0.79	11.38	7.59	7.65
Quintile 5	3703	7.94	4.35	11.39	7.91	7.98
Non-working age pop						
Quintile 1	3728	8.40	1.27	11.38	8.37	8.42
Quintile 2	3723	8.06	0.19	11.39	8.03	8.09
Quintile 3	3739	7.85	1.12	11.38	7.82	7.89
Quintile 4	3714	7.75	0.15	11.38	7.72	7.78
Quintile 5	3726	7.68	0.79	11.37	7.64	7.72
Non-owner occ. housing						
Quintile 1	3728	7.66	0.79	11.37	7.63	7.69
Quintile 2	3745	7.69	0.18	11.33	7.66	7.72
Quintile 3	3708	7.94	0.15	11.39	7.91	7.98
Quintile 4	3727	8.08	1.27	11.39	8.05	8.12
Quintile 5	3722	8.36	1.00	11.38	8.33	8.39
Non-white ethnicity						
Quintile 1	3730	7.46	0.18	11.39	7.43	7.49
Quintile 2	3728	7.68	0.15	11.37	7.64	7.71
Quintile 3	3722	7.93	0.19	11.37	7.89	7.96
Quintile 4	3725	8.22	1.02	11.39	8.19	8.25
Quintile 5	3725	8.46	1.71	11.38	8.43	8.48
Non-prof socioecon. group						
Quintile 1	3727	8.31	0.15	11.38	8.29	8.34
Quintile 2	3727	8.00	1.27	11.37	7.97	8.03
Quintile 3	3782	7.78	3.46	11.38	7.75	7.81
Quintile 4	3669	7.69	0.79	11.37	7.66	7.73
Quintile 5	3725	7.95	3.11	11.39	7.91	7.98
Unskilled social group						
Quintile 1	3727	8.26	0.15	11.36	8.23	8.29
Quintile 2	3731	7.87	0.19	11.37	7.84	7.90
Quintile 3	3722	7.73	3.18	11.30	7.70	7.76
Quintile 4	3726	7.73	3.59	11.33	7.70	7.76
Quintile 5	3724	8.15	3.11	11.39	8.11	8.18
Unemployed						
Quintile 1	3732	8.06	0.18	11.37	8.04	8.09
Quintile 2	3726	7.91	0.15	11.36	7.88	7.94
Quintile 3	3733	7.84	0.19	11.36	7.81	7.88
Quintile 4	3742	7.85	3.91	11.37	7.81	7.88
Quintile 5	3697	8.07	3.11	11.39	8.04	8.11

**Table 5:** Descriptive statistics by rural quintiles

Variable	N	Mean	Min	Max	Lower 95% CI	Upper 95% CI
HP Index 2016						
Quintile 1	1071	6.83	4.35	11.34	6.77	6.88
Quintile 2	1954	7.09	4.70	11.29	7.05	7.12
Quintile 3	2077	7.34	3.18	11.36	7.30	7.37
Quintile 4	1596	7.55	5.15	11.01	7.51	7.59
Quintile 5	524	7.73	5.21	11.37	7.67	7.80
No third-level education						
Quintile 1	237	7.62	5.21	10.61	7.51	7.74
Quintile 2	1381	7.53	3.18	11.37	7.48	7.58
Quintile 3	2163	7.32	4.70	11.30	7.29	7.36
Quintile 4	2252	7.19	4.36	11.29	7.16	7.22
Quintile 5	1230	6.99	4.35	11.34	6.94	7.04
Non-working age pop						
Quintile 1	256	7.35	4.36	10.25	7.23	7.46
Quintile 2	1239	7.41	4.81	11.36	7.37	7.46
Quintile 3	1811	7.38	4.86	11.37	7.34	7.42
Quintile 4	2067	7.30	4.35	11.30	7.26	7.34
Quintile 5	1890	7.05	3.18	11.34	7.00	7.09
Non-owner occ. housing						
Quintile 1	2649	7.35	4.36	11.37	7.32	7.39
Quintile 2	2369	7.26	4.35	11.33	7.23	7.30
Quintile 3	1282	7.16	3.18	11.36	7.11	7.21
Quintile 4	735	7.24	4.67	10.75	7.18	7.30
Quintile 5	228	7.21	5.24	11.34	7.10	7.32
Non-white ethnicity						
Quintile 1	2982	7.23	3.18	11.30	7.19	7.26
Quintile 2	2331	7.31	4.36	11.37	7.28	7.35
Quintile 3	1391	7.29	4.73	10.61	7.24	7.33
Quintile 4	468	7.31	4.67	10.33	7.23	7.38
Quintile 5	91	7.45	5.25	9.77	7.27	7.63
Non-prof socioecon. group						
Quintile 1	750	7.78	5.21	10.98	7.73	7.84
Quintile 2	1724	7.57	4.36	11.37	7.53	7.61
Quintile 3	1982	7.30	4.67	11.34	7.27	7.34
Quintile 4	1745	7.06	3.18	11.14	7.02	7.10
Quintile 5	1062	6.73	4.35	9.56	6.68	6.78
Unskilled social group						
Quintile 1	940	7.72	5.38	10.98	7.67	7.77
Quintile 2	2053	7.46	5.05	11.37	7.42	7.49
Quintile 3	2024	7.23	3.18	11.30	7.19	7.26
Quintile 4	1586	6.99	4.36	11.33	6.94	7.03
Quintile 5	660	6.90	4.35	11.34	6.83	6.98
Unemployed						
Quintile 1	1479	7.51	5.15	11.37	7.47	7.55
Quintile 2	1796	7.41	4.87	11.07	7.37	7.45
Quintile 3	1741	7.25	3.18	11.36	7.21	7.29
Quintile 4	1468	7.09	4.35	11.33	7.04	7.13
Quintile 5	779	6.92	4.73	11.34	6.85	6.99

**Table 6:** Descriptive Statistics by urban quintiles

Variable	N	Mean	Min	Max	Lower 95% CI	Upper 95% CI
HP Index 2016						
Quintile 1	2585	8.38	5.82	11.39	8.35	8.42
Quintile 2	1729	8.29	0.79	11.38	8.25	8.34
Quintile 3	1605	8.30	0.19	11.02	8.26	8.34
Quintile 4	2069	8.35	1.00	11.36	8.31	8.39
Quintile 5	3085	8.46	0.15	11.00	8.44	8.49
No third-level education						
Quintile 1	3487	8.49	0.15	11.02	8.46	8.51
Quintile 2	2347	8.32	1.00	11.02	8.28	8.35
Quintile 3	1563	8.25	0.19	11.36	8.21	8.29
Quintile 4	1492	8.27	0.79	11.38	8.23	8.32
Quintile 5	2473	8.42	5.82	11.39	8.38	8.45
Non-working age pop						
Quintile 1	3472	8.47	1.27	11.38	8.45	8.50
Quintile 2	2484	8.38	0.19	11.39	8.35	8.42
Quintile 3	1928	8.30	1.12	11.38	8.26	8.34
Quintile 4	1647	8.31	0.15	11.38	8.27	8.36
Quintile 5	1836	8.33	0.79	11.37	8.29	8.37
Non-owner occ. housing						
Quintile 1	1079	8.42	0.79	11.36	8.38	8.46
Quintile 2	1376	8.43	0.18	11.01	8.39	8.47
Quintile 3	2426	8.36	0.15	11.39	8.32	8.39
Quintile 4	2992	8.29	1.27	11.39	8.26	8.32
Quintile 5	3494	8.43	1.00	11.38	8.41	8.46
Non-white ethnicity						
Quintile 1	748	8.39	0.18	11.39	8.32	8.45
Quintile 2	1397	8.28	0.15	11.37	8.23	8.33
Quintile 3	2331	8.31	0.19	11.37	8.27	8.34
Quintile 4	3257	8.35	1.02	11.39	8.33	8.38
Quintile 5	3634	8.48	1.71	11.38	8.45	8.51
Non-prof socioecon. group						
Quintile 1	2977	8.45	0.15	11.38	8.42	8.48
Quintile 2	2003	8.37	1.27	11.36	8.33	8.41
Quintile 3	1800	8.31	3.46	11.38	8.27	8.35
Quintile 4	1924	8.27	0.79	11.37	8.23	8.31
Quintile 5	2663	8.43	3.11	11.39	8.39	8.47
Unskilled social group						
Quintile 1	2787	8.44	0.15	11.36	8.41	8.47
Quintile 2	1678	8.37	0.19	11.02	8.32	8.41
Quintile 3	1698	8.34	3.91	11.02	8.30	8.38
Quintile 4	2140	8.28	3.59	11.01	8.24	8.32
Quintile 5	3064	8.41	3.11	11.39	8.38	8.45
Unemployed						
Quintile 1	2253	8.43	0.18	10.98	8.40	8.46
Quintile 2	1930	8.37	0.15	11.36	8.34	8.41
Quintile 3	1992	8.36	0.19	11.01	8.32	8.40
Quintile 4	2274	8.34	3.91	11.37	8.31	8.38
Quintile 5	2918	8.38	3.11	11.39	8.35	8.41

## Footnotes

1 The Irish Census is collected once every five years. Due to the Covid-19 pandemic, the 2021 census was delayed, resulting in the 2016 SA data being the most recent available.

2 For a comprehensive overview of the DIMAQ model see Shaddick et al. [43].

3 For more information on the specifics of how the Haase-Pratschke Deprivation Index is calculated, visit: https://www.pobal.ie/research-analysis/.

4 There were 12 small areas which did not have corresponding PM_2*.*5_ data so were dropped from the analysis.

5 (Tables 4, 5 and 6 in Appendix C provide additional information on the ranges and 95% Confidence Intervals).

6 Concentration indices were also calculated for small areas stratified by urban and rural areas, which were all very small in magnitude. However, slightly more inequality towards more disadvantaged areas was observed in both 2011 and 2016. These are available on request from the authors.
